# Block Copolymer Modified Nanonetwork Epoxy Resin for Superior Energy Dissipation

**DOI:** 10.3390/polym14091891

**Published:** 2022-05-05

**Authors:** Suhail K. Siddique, Hassan Sadek, Tsung-Lun Lee, Cheng-Yuan Tsai, Shou-Yi Chang, Hsin-Hsien Tsai, Te-Shun Lin, Gkreti-Maria Manesi, Apostolos Avgeropoulos, Rong-Ming Ho

**Affiliations:** 1 Department of Chemical Engineering, National Tsing Hua University, Hsinchu 30013, Taiwan; s105032891@m105.nthu.edu.tw (S.K.S.); hassan_sadek@azhar.edu.eg (H.S.); tsunglunlee@mx.nthu.edu.tw (T.-L.L.); 2 Department of Material Science and Engineering, National Tsing Hua University, Hsinchu 30013, Taiwan; tsai10681@gmail.com (C.-Y.T.); changsy@mx.nthu.edu.tw (S.-Y.C.); 3 Kaohsiung Factory R&D Department, Chang Chun Plastics Co., Ltd., Kaohsiung 81469, Taiwan; hsin_hsien_tsai@ccpgp.com (H.-H.T.); de_shun_lin@ccpgp.com (T.-S.L.); 4 Department of Materials Science Engineering, University Campus, University of Ioannina, 45110 Ioannina, Greece; gretimanesi@uoi.gr (G.-M.M.); aavger@uoi.gr (A.A.); 5 Faculty of Chemistry, Lomonosov Moscow State University (MSU), GSP-1, 1-3 Leninskiye Gory, 119991 Moscow, Russia

**Keywords:** nanonetwork, block copolymer, modifier, templated polymerization, energy dissipation

## Abstract

Herein, this work aims to fabricate well-ordered nanonetwork epoxy resin modified with poly(butyl acrylate)-*b*-poly(methyl methacrylate) (PBA-*b*-PMMA) block copolymer (BCP) for enhanced energy dissipation using a self-assembled diblock copolymer of polystyrene-*b*-poly(dimethylsiloxane) (PS-*b*-PDMS) with gyroid and diamond structures as templates. A systematic study of mechanical properties using nanoindentation of epoxy resin with gyroid- and diamond-structures after modification revealed significant enhancement in energy dissipation, with the values of 0.36 ± 0.02 nJ (gyroid) and 0.43 ± 0.03 nJ (diamond), respectively, when compared to intrinsic epoxy resin (approximately 0.02 ± 0.002 nJ) with brittle characteristics. This enhanced property is attributed to the synergic effect of the deliberate structure with well-ordered nanonetwork texture and the toughening of BCP-based modifiers at the molecular level. In addition to the deliberate structural effect from the nanonetwork texture, the BCP modifier composed of epoxy-philic hard segment and epoxy-phobic soft segment led to dispersed soft-segment domains in the nanonetwork-structured epoxy matrix with superior interfacial strength for the enhancement of applied energy dissipation.

## 1. Introduction

Epoxy resin is one of the most versatile thermosets due to its processability, cost-effectiveness, and superior mechanical performance, giving excellent characteristics for industrial applications. The existence of multiple oxiranes or epoxy groups in the molecular structure of epoxy resin can provide high crosslinking density for superior mechanical strength, but the excessive crosslinking of epoxy rings in the molecular structure results in the brittle or glassy characteristics, and thus limits their engineering performance [1]. As a result, the reduction in glassy characteristics of epoxy resins to enhance the toughness gained intense attention in the last century. Extensive research has been conducted to improve the energy absorption capability of epoxy resin by incorporating a variety of toughening agents including liquid rubber [2,3], thermoplastic polymers [4], core–shell particle [5,6], and ceramic fillers [7].

A well-dispersed rubber modifier without any molecular level interactions can significantly reduce the brittle characteristics of epoxy resin by lowering the crosslinking density, which enhances their toughness by enabling the internal cavitation of well-bounded rubber particles [8]. However, it might greatly affect their strength and glass transition temperature (T_g_) [1]. Recent developments in the toughening of epoxy resin using amphiphilic block copolymers was reported [9,10] at which the BCPs self-assemble into spherical micelle, worm-like structures or vesicles to provide high energy dissipation with the least impact on modulus and glass transition temperature [11]. The BCPs composed of epoxy-philic hard and epoxy-phobic soft segments can self-assemble into different morphologies in epoxy resin due to their intermolecular interactions, which gives them the exceptional capability to absorb applied energy through cavitation with shear banding. The addition of a small amount of BCPs provides excellent toughening without sacrificing the strength and effective thermal properties of epoxy resin [8,12]. Most interestingly, the energy dissipation from the brittle to plastic transformation of intrinsic epoxy resin can also be achieved by well-ordered structures termed metamaterials [13,14,15], which offer unique emergent mechanical properties, especially for high specific energy absorption [16,17,18,19]. Mechanical metamaterials are the materials that show exceptional mechanical properties due to their deliberate structuring instead of bulk behavior [19].

Moreover, nanonetwork materials have been reported for their enhanced mechanical properties due to their network structure in the nanoscale, where the nanosize can be the secondary aspect of the mechanical property [20]. However, the fabrication of nanonetwork structures is extremely challenging due to the difficulty of controlling their structure. The block copolymers have been extensively studied recently due to their ability to self-assemble into various periodic nanostructures depending on the volume fraction of their constituent segments and molecular weight [21,22,23,24]. Due to their unique network geometry, gyroid and diamond phases are considered appealing morphologies for practical applications [25,26]. By taking advantage of the degradable segments in BCPs, polymeric templates with well-ordered periodic nanochannels can be fabricated and subsequently serve as a template for templated syntheses, giving a platform technology for the fabrication of nanonetwork functional materials [27]. The templated syntheses can be carried out by atomic layer deposition [28], electroless plating [29], sol–gel reaction [30], electrochemical deposition [31] and templated polymerization [32]. Recently, the enhanced energy dissipation from the deliberate structuring of nanonetwork textures for thermosets fabricated by templated polymerization has been demonstrated by enabling the design of mechanical metamaterials from a bottom-up approach [33].

**Figure 1:**
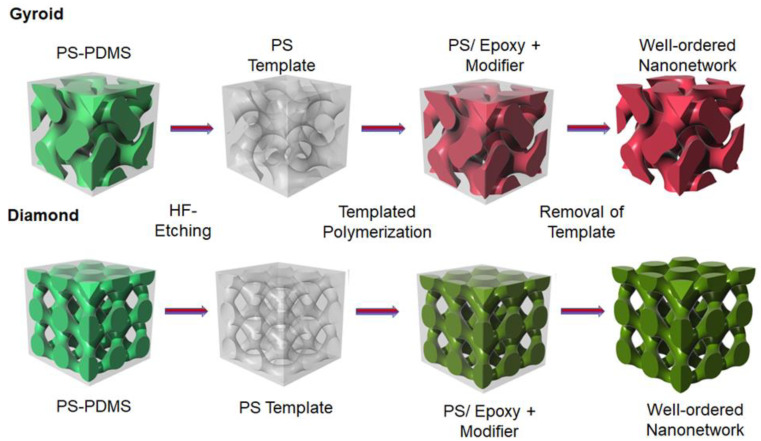
Schematic illustration of templated syntheses of epoxy resin modified by PBA-*b*-PMMA.

Herein, this work aims to demonstrate the fabrication of poly(butyl acrylate)-*b*-poly(methyl methacrylate) (PBA-*b*-PMMA) modified epoxy resin with well-ordered nanonetwork structures for the enhancement of energy dissipation capability using the self-assembled BCP, followed by templated polymerization. As shown in Figure 1, a well-ordered gyroid and diamond phase with co-continuous PS and PDMS domains from self-assembly of a polystyrene-*b*-poly(dimethylsiloxane) can be fabricated by solution casting using selective solvents. Selective etching of the PDMS block from PS-*b*-PDMS can be acquired using hydrofluoric (HF) acid to give nanoporous PS with gyroid- and diamond-structured nanochannels as templates for polymerization of modified epoxy resin. The examination of the mechanical energy dissipation of these two distinct nanonetwork epoxy resins after the modification with PBA-*b*-PMMA has been carried out by using nanoindentation; the PBA-*b*-PMMA BCP can be self-assembled into spherical nanosized micelle in the epoxy matrix which acts as a toughening agent for the formation of soft domains. The synergic effect of the deliberate structuring in nanoscale and the toughening of the BCP-based modifier on a molecular level can significantly contribute to the energy dissipation capability of nanonetwork epoxy resins as compared to intrinsic brittle epoxy resin.

## 2. Materials and Methods

The detailed synthesis procedure for PS-*b*-PDMS was discussed previously [34,35]. Epoxy resin used in this study is Bisphenol-A diglycedyle ether (DGEBA) (DEH 24, Dow Chemical, Midland, MI, USA) and the hardener is Triethylenetetramine (TETA) (DEH 24, Dow Chemical). Solvents used in this study is toluene (Sigma Aldrich, St. Louis, MI, USA) and chloroform (Sigma Aldrich).

### 2.1. Synthesis Procedures

The total number average molecular weight of the PS-*b*-PDMS used in this study was 86,000 g/mol (${\bar{M}}_{n}^{PS\;}$: 51,000 g/mol; ${\bar{M}}_{n}^{PDMS\;}$: 35,000 g/mol) with the volume fraction of PDMS equal to 0.42, giving lamellar morphology during self-assembly using a neutral solvent. The dispersity values of the PS precursor and the final synthesized copolymer is described in Table 1.

The gyroid and diamond-structures in the PS-*b*-PDMS can be fabricated by solution casting in PS selective solvents such as toluene and chloroform, respectively.

### 2.2. Preparation of Well-Ordered Nanoporous Template

The lamellae-forming PS-*b*-PDMS was dissolved in PS selective solvents, including toluene (Sigma Aldrich) and chloroform (Sigma Aldrich) (10 wt% concentration), in a vial with a controlled solvent evaporation rate. After drying, the samples were further dried at 60 °C in a vacuum oven. The formation of network phases from the self-assembly of lamellae-forming PS-*b*-PDMS indicates that a double gyroid structure can be formed using toluene for solution casting while a double diamond structure can be formed using chloroform as a solvent. Moreover, the affinity of PS towards the PS-selective solvents induces a reduction in the PDMS volume fraction causing the flat interfaces (lamellar morphology) to generate network or cylindrical structures. For toluene (δ_toluene_ = 8.9 cal^1/2^/cm^3/2^), the difference in the solubility parameters (δ_PS_ = 9.1 cal^1/2^/cm^3/2^, δ_PDMS_ = 7.4 cal^1/2^/cm^3/2^) indicates selectivity towards PS domains, giving rise to double gyroid. Furthermore, chloroform (δ_chloroform_ = 9.3 cal^1/2^/cm^3/2^) enables the formation of different network phases such as double diamond and double primitive (DP), as already reported in the literature [36]. Note that PDMS swollen ratio is 10% larger in chloroform than in toluene, as reported by Whitesides et al. [37]. Accordingly, higher elasticity and free stretching energy provided by the PDMS segments can contribute to the formation of kinetically trapped phases. This behavior may be attributed to PDMS blocks expansion (instead of looping) into the core of the junctions, even with the entropy loss at a higher strut number. As a result, network textures such as double diamond and double primitive can be formed due to the effect of evaporation rate control on solution casting. Subsequently, the selective etching between the PS and PDMS segments allows the formation of the nanoporous PS template from the self-assembled PS-*b*-PDMS using HF solution (HF/H_2_O/methanol = 0.5/1/1 by volume). After the complete removal of the PDMS followed by washing with water and methanol, well-ordered nanoporous PS templates with gyroid- and diamond-structures with the corresponding nanochannels (approximately equivalent porosity) for templated polymerization can be obtained.

### 2.3. Templated Polymerization

For templated polymerization of epoxy resin, successful pore-filling can be achieved using methanol combined with an epoxy resin precursor for high wetting ability. The hydrophobic inner walls of the PS template can be effectively prepared for pore filling using short-chain alcohols. Note that effective pore filling must be confirmed before templated polymerization of epoxy resin; if polymerization starts before pore filling it can cause blocking of the template, which leads to incomplete networks. A mixture of dissolved epoxy resin containing Bisphenol-A type epoxy (DER 331, Dow Chemical) with 5% wt% of well soluble PBA-*b*-PMMA (see Table S1 for details) and triethylenetetramine (TETA) (DEH 24, Dow Chemical) was initially prepared. The PS templates were immersed into the precursor solution at low temperature (10 °C) for five hours to reduce the polymerization reaction and promote adequate pore filling. The epoxy resin can be pore-filled into the nano channeled templates by capillary force. The mild curing of epoxy resin inside the template gradually leads to an insufficient cross-linking reaction of the resin. A temperature increase can increase the crosslinking, but it might damage the template texture. To solve specific issues multistep curing was conducted. The temperature was gradually raised to the final setting temperature for curing through stepwise heating to provide the optimum temperature. Consequently, a higher degree of curing was acquired. Direct heating from room temperature to 150 °C led to template damage with disordered texture for templated resin. The high internal stress triggered by the fast heating causes the deformation of the resin skeleton.

### 2.4. Transmission Electron Microscopy (TEM)

Bright-field (BF) transmission electron microscopy (TEM) imaging was used to determine the pore-filling of epoxy resin in the PS template using JEOL JEM-2100 LaB_6_ (Akishima, Tokyo, Japan) at an accelerating voltage of 200 kV by mass thickness contrast.

### 2.5. Field-Emission Scanning Electron Microscopy (FESEM)

Field emission scanning electron microscopy (FESEM) observation was performed at an accelerating voltage of 5 keV on a JEOL JSM-7401F (Akishima). The samples were collected on a silicon wafer and sputter-coated with platinum at approximately 2 to 3 nm.

### 2.6. Small-Angle X-ray Scattering (SAXS)

National Synchrotron Radiation Research Center (NSRRC) with synchrotron X-ray beamline X27C was used to study the SAXS where the wavelength of the beam was 0.155 nm. The two-dimensional SAXS pattern was obtained using the MAR CCD X-ray detector (Rayonix L.L.C., Evanston, IL, USA), at which the (1D) linear profile was obtained by integration of the 2D pattern. The scattering angle of the SAXS pattern was calibrated using silver behenate with the first order scattering vector q*(4π sin θ)/λ, where 2θ is the scattering angle).

### 2.7. Nanoindentation Measurements

Hysitron Ti950 triboindenter (Hysitron Inc. Minneapolis, MN, USA) was used to perform the nanoindentation tests using a spherical indenter with a 2 µm diameter. The indentation measurements were conducted on a microtome film sample with 5 µm thickness in a silicon wafer as a substrate at room temperature. The load–displacement curve was recorded at the same rate of loading and unloading (60 μN/s) with a maximum load of 500 μN applied. In the nanoindentation tests, the load–displacement data were recorded continuously, while the tip was driven into the composite materials, and then smoothly removed. The load–displacement (L-D) curves were then used to calculate the mechanical energy dissipation of the fabricated materials at the same rate of loading and unloading (60 μN/s). For nanoindentation, the mechanical properties of reduced elastic modulus and hardness can be calculated from the load–displacement curve (P-h) based on the widely used Oliver-Pharr model. In the present study, the reduced elastic modulus *E_r_* was determined from the P h curve, using the Sneddon formula for spherical indenter frictionless punch.

(1) $$Er=\frac{\surd \pi }{2}\frac{S}{\surd A_{t}}$$

Here, *E_r_* is the reduced elastic modulus (indentation modulus) which represents the elastic deformation that occurs in both the sample and indenter tip. *S* is stiffness. *A_t_* represents the projected contact area. Note that the deformation in the diamond indenter tip is negligible. As a result, the reduced elastic modulus (indentation modulus) is a representative value for the discussion with respect to mechanical performance.

## 3. Results and Discussion

**Figure 2:**
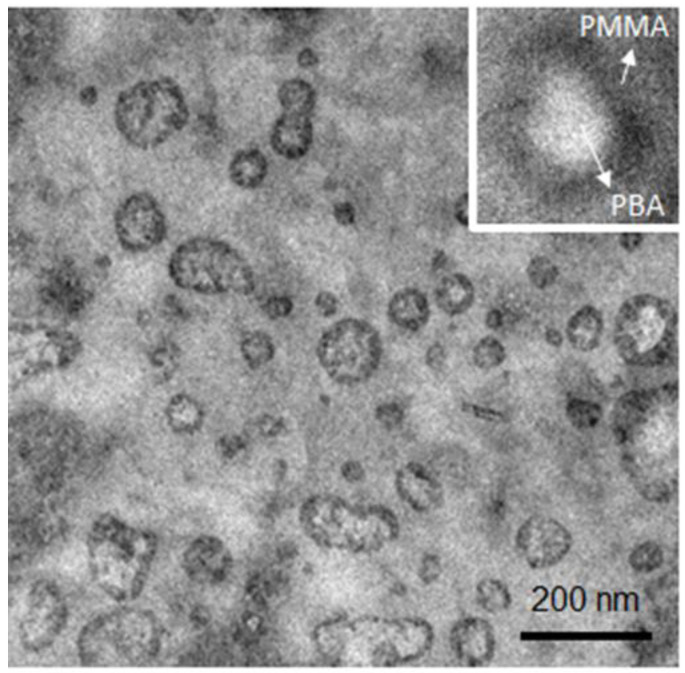
TEM micrograph of epoxy resin modified with 5% *w*/*w* of PBA-*b*-PMMA where the inset corresponds to a magnified image of the self-assembled PBA-*b*-PMMA and identifies the two different blocks. Vapor staining with 0.5 wt% OsO_4_ aqueous solutions for 24 h at ambient temperature was performed leading to dark (PMMA) and bright (PBA) contrast.

**Figure 3:**
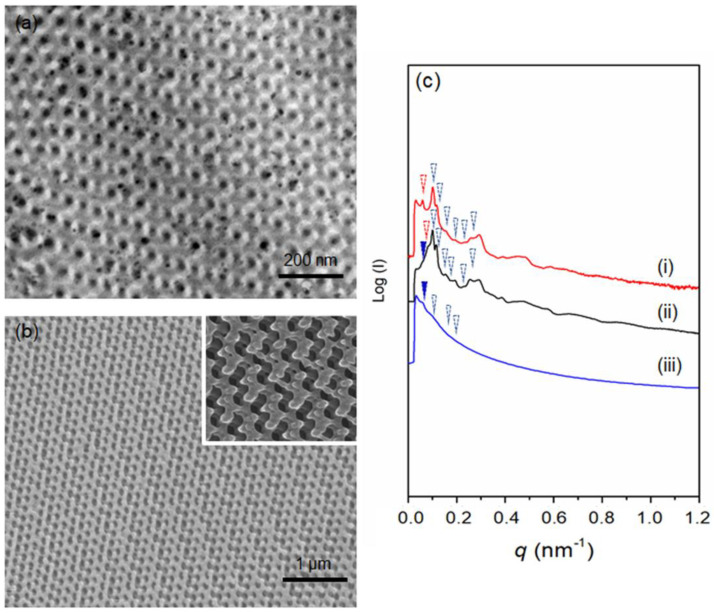
(**a**) TEM image of PS/epoxy nanocomposite in gyroid structure. (**b**) FESEM image of well-ordered gyroid epoxy resin, inset shows the magnified image. (**c**) 1D SAXS profiles of (i) PS template, (ii) PS/epoxy nanocomposites, and (iii) nanoporous epoxy resin with gyroid texture.

The study of the toughening mechanism by adding PBA-*b*-PMMA to brittle material such as epoxy resin starts from the basic characterization of the modifier (PBA-*b*-PMMA) (The detailed information about the sample is described in Table S1). As shown in Figure S1a, the glass transition temperature (T_g_) of the soft PBA segment is approximately −35 °C, whereas the hard PMMA shows T_g_ at 87 °C (Figure S1b). Note that the T_g_ of the acrylic containing soft segment is significant for mechanical stability in bisphenol A diglycedyle ether (DGEBA) type of epoxy resin to achieve the desired toughness. The hard segment (PMMA) was expected to provide the molecular level association with epoxy resin in the nanoscale. Moreover, the T_g_ of the soft segment (PBA) was reaffirmed by DMA analysis, as shown in Figure S1c where the tan δ peak appears at approximately −37 °C. After the introduction of the PBA-*b*-PMMA modifier to the epoxy resin, the feasibility of reaction-induced phase separation was evaluated using TEM analysis of the dispersed PBA-*b*-PMMA in the epoxy matrix. As shown in Figure 2, the PBA-*b*-PMMA can be self-assembled into a spherical micelle structure dispersed in the epoxy matrix. The samples were vapor stained with 0.5 wt% OsO_4_ aqueous solutions for 24 h at ambient temperature to provide adequate contrast between the two segments. In Figure 2 inset, the PBA microdomain appears as bright and PMMA as dark in a grey epoxy resin matrix, suggesting the formation of compatible PMMA in DGEBA. Note that PBA serves as the soft segment core in the dispersed micelle structure and PMMA act as the shell. Moreover, the spherical PBA-*b*-PMMA domains act as an impact modifier due to the compatibility between epoxy and PMMA, based on their interfacial strength. Namely, the incompatible PBA block dispersed in the epoxy resin matrix can be characterized as an epoxy-phobic core and the compatible PMMA block as an epoxy-philic shell that led to the reinforcement of the interfacial strength. Additionally, as shown in Figure S1d, the glass transition temperature of the well-cured intrinsic epoxy resin was approximately 84 °C, whereas the addition of epoxy resin modified with 5% PBA-*b*-PMMA reduces the T_g_ to 83 °C; note that the reduction in T_g_ with respect to the addition of a modifier was negligible. After the gradual addition of PBA-*b*-PMMA up to 20% *w*/*w*, there is an obvious reduction in the T_g_ to 73 °C. Following the platform technology developed in our laboratory, the fabrication of well-ordered nanonetwork epoxy modified with PBA-*b*-PMMA can be successfully achieved using templated polymerization. By taking advantage of the strong segregation strength between PS and PDMS, gyroid-structured PS-*b*-PDMS can be fabricated from lamellae-forming BCP using toluene as a selective solvent [34]. Table 1 summarizes the molecular characterization details of the PS-*b*-PDMS used in this study. Figure S2a shows the TEM projection of the solution-cast PS-*b*-PDMS; a typical projection of a trigonal planar gyroid phase can be observed. The 1D SAXS results at the relative *q* values of √6, √8, √16, √22, √38, and √52 (Figure S2b) indicate the double gyroid phase (space group of *Ia*$\bar{3}$*d*). By using chloroform as a selective solvent, a typical projection of a double diamond phase with a tetrapod-like pattern can be identified by TEM analysis (Figure S2c). The corresponding 1D SAXS profile with relative *q* values of √2, √3, √6, √10, √18 and √20 (Figure S2d) are in good agreement with the double diamond phase in the *Pn*$\bar{3}$*m* space group [38]. Following the experimental procedures shown in Figure 1, the pore-filling of the PBA-*b*-PMMA modified epoxy resin and thermal treatment at 110 °C and 150 °C for effective crosslinking of the precursor of epoxy resin can be carried out to produce PS/epoxy nanocomposites through templated polymerization. The TEM micrograph in Figure 3a shows the PS matrix as bright domain and the epoxy as dark domain due to the effective staining using OsO_4_, which further confirms the formation of a gyroid-structured PS/epoxy nanocomposite. Subsequently, after removal of the PS template using organic solvents such as styrene monomer, well-ordered gyroid-structured epoxy resin can be obtained, as evidenced by FESEM (Figure 3b). Moreover, the one-dimensional small-angle X-ray scattering (1D SAXS) profile reaffirms the observed morphology.

**Figure 4:**
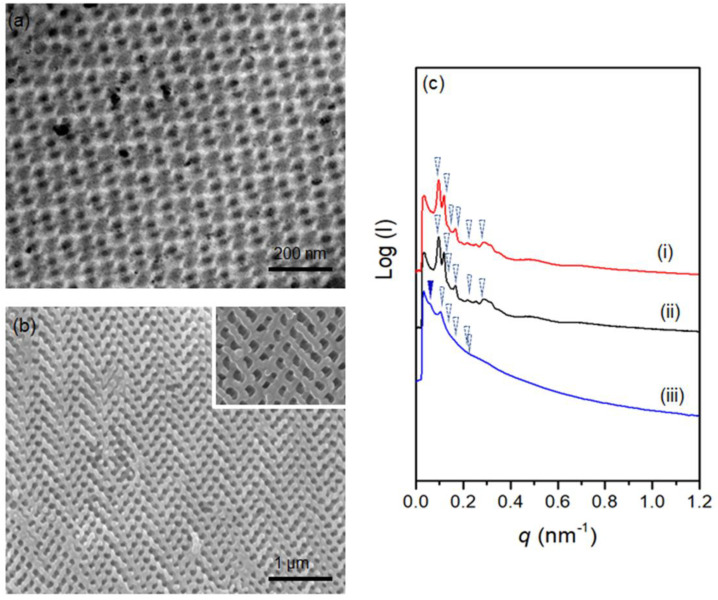
(**a**) TEM image of PS/epoxy nanocomposite in the diamond structure. (**b**) FESEM image of well-ordered diamond epoxy resin, inset shows the magnified image. (**c**) 1D SAXS profiles of (i) PS template, (ii) PS/epoxy nanocomposites, and (iii) nanoporous epoxy resin with diamond structure.

**Figure 5:**
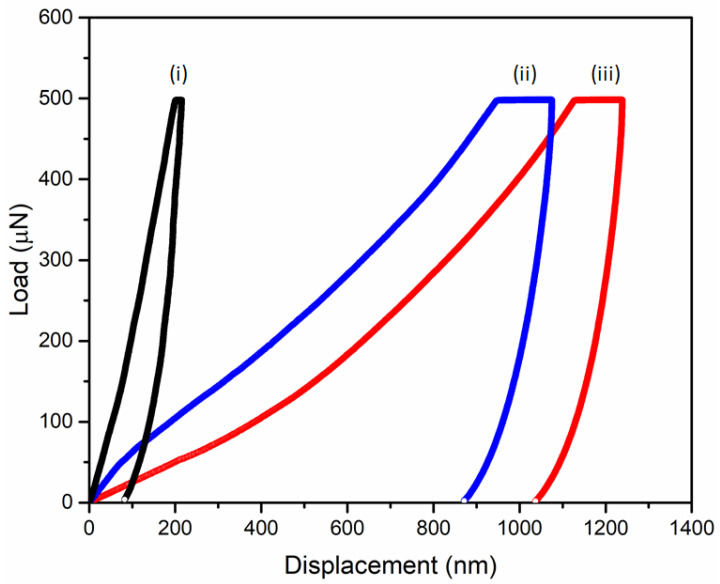
Load–displacement tests of (i) non-structured epoxy without a modifier, (ii) gyroid-structured epoxy, and (iii) diamond-structured epoxy resin with PBA-*b*-PMMA under 500 μN.

As shown in Figure 3c(i), the characteristic reflections corresponding to the 1D SAXS results with the relative *q* values of √6, √8, √16, √20, √30, and √38 and an additional reflection at √4 are recognized as the double gyroid phase (blue arrow) with an additional slight deformation peak (denoted by red arrow). The appearance of an extra peak at a relative *q* value of √2 can be observed for PS/epoxy nanocomposite in Figure 3c(ii), which is attributed to the (dark blue) network shifting of the gyroid nanonetworks during templated polymerization [39,40]. After the removal of the PS template, the shifting is more significant [Figure 3c(iii)]. Correspondingly, diamond-structured epoxy resin can be obtained by following the same experimental procedure. As shown in Figure 4a, the TEM projection verifies the formation of diamond network-structured PS/epoxy nanocomposites (See Figure S3 for details). Subsequently, after the removal of the PS template, nanonetwork-structured epoxy can be acquired, as evidenced by FESEM results in Figure 4b. As shown in Figure 4c(i,ii), a set of characteristic reflections for the double diamond phase at the relative *q* values of √2, √3, √4, √6, √8, and √10 are identified for the PS template and PS/epoxy nanocomposites. Consequently, a characteristic reflection of the nanoporous epoxy at the relative *q* value of √3 can be found in Figure 4c(iii) due to the network shifting after the removal of the template. The above-mentioned SAXS results (Figure 4c) confirm the successful fabrication of diamond-structured modified resin. The mechanical properties of the well-ordered nanonetwork thermosets were investigated using nanoindentation analysis. Figure 5 shows a typical load–displacement curve for intrinsic epoxy resin, as well as gyroid- and diamond-structured epoxy resins modified with PBA-*b*-PMMA. Based on the unloading curve, the reduced elastic modulus (*E_r_*) of 4.2 GPa was calculated for intrinsic epoxy resin by the Oliver-Pharr model [41]; note that the epoxy resins are inherently brittle due to their highly cross-linked structure at which the unloading curve retracts predominantly to the initial state due to the elastic behavior. Interestingly, the modified epoxy resin with PBA-*b*-PMMA shows approximately 90% of plastic deformation behavior with minor retracting of the unloading curve (Figure S4). The reduced elastic modulus of epoxy resin (without deliberate structuring) after the addition of PBA-*b*-PMMA is approximately 3.8 GPa, indicating that there is no significant effect on the modulus after adding the modifier. In contrast to the intrinsic epoxy resin, a reduction in the modulus to 0.9 GPa for the gyroid-structured nanonetwork epoxy resin due to the introduction of porous texture. Consistently, the diamond-structured epoxy resin shows a lower modulus with a value of 0.8 GPa. It is important to note that acrylate-base block copolymer has better thermal and oxidative stability which prevents degradation during high-temperature curing (Figure S1d) due to the compatibility of PMMA towards epoxy. As a result, the dispersion of PBA-*b*-PMMA spherical domains in the matrix of epoxy resin enables better mechanical properties without compromising the thermal characteristics. For the energy dissipation measurement, the area enclosed by loading and unloading curves was calculated. For homogeneous non-structured epoxy resin, the calculated energy dissipation by area under the load–displacement curve is 0.02 ± 0.002 nJ; note that the epoxy resin with the highest crosslinking density are nearly 90% elastic at 500 µN load (Figure S4). By contrast, after being modified with 5% *w*/*w* of PBA-*b*-PMMA, a five-times higher energy dissipation can be observed which equals to 0.09 ± 0.004 nJ, which is much higher than the intrinsic brittle epoxy resin due to the plastic mode of deformation. This enhanced energy dissipation might be attributed to the stress concentration on the PBA soft segment, where the rubbery core of the self-assembled PBA-*b*-PMMA leads to a cavitate inside the epoxy matrix with shear band yielding, which accounts for the enhanced toughening. Note that the PBA in the BCP modifier incompatible to DGEBA epoxy resin that acts as a core in the self-assembled spherical micelle, where the compatibility between PMMA and epoxy allows the intermolecular interaction between the epoxy resin and the BCP-based modifier. As a result, the compatibility of PBA-*b*-PMMA controls the reduction in modulus, whereas the soft segment can enhance the energy dissipation. Most interestingly, the energy dissipation from brittle to the plastic deformation of intrinsic epoxy resin can be further enhanced by using artificially engineered structures. For gyroid-structured epoxy resin, the energy dissipation was calculated to 0.36 ± 0.02 nJ, a value quite higher than the one obtained for intrinsic epoxy resin. In contrast to gyroid-structured epoxy resin, diamond-structured epoxy resin show a large energy dissipation at a given loading with less retracting for the unloading (Figure 5); both gyroid and diamond structures give the deliberate structuring effect on energy dissipation, at which well-ordered nanonetwork structures can enhance the energy dissipation value up to six and eight times (Figure S5), compared to the intrinsic epoxy as reported in our previous publication [33]. These superior enhancements in mechanical energy dissipation is attributed to the well-ordered nanonetworks in gyroid and diamond structure with isotropic periodicity plastic deformation. Moreover, the higher strut number in the diamond network in comparison with the triagonal planar gyroid network justifies the additional energy dissipation along the struts equally and symmetrically. As a result, a recognizable increase in the energy dissipation can be found in the PBA-*b*-PMMA modified diamond epoxy resin with a value of 0.43 ± 0.03 nJ (more than twenty (20) times of the non-structured, intrinsic epoxy resin). The enhancement of the energy dissipation explicitly indicates the synergic effect of the deliberate structuring of network texture in the nanoscale and the toughening of self-assembled modifiers (BCPs) in the epoxy matrix at the molecular level.

## 4. Conclusions

In conclusion, well-ordered nanonetwork-structured epoxy resin with gyroid (trigonal planar) and diamond (tetrapod) structures modified with a BCP based modifier, PBA-*b*-PMMA, can be successfully fabricated by templated polymerization, using PS templates. The periodic structured templates were acquired from PS-*b*-PDMS self-assembled samples, followed by preferential removal of PDMS through HF etching. The incorporation of PBA-*b*-PMMA in the matrix of epoxy resin imparts self-assembled spherical micelles. The PBA core serves as an energy absorbing soft domain due to the core–shell characteristics with reinforced interfacial strength from the association of PMMA in the matrix of epoxy resin. By taking advantage of the well-ordered nanonetwork structure, a further enhancement on plasticity can be achieved. The synergic effect of the deliberate structuring of nanonetwork texture and the toughening of the BCP-based modifier, thus, presented outstanding enhancement of plastic energy dissipation for gyroid and diamond-structured resin.

## Figures and Tables

**Table 1:** Molecular characterization of the synthesized PS-*b*-PDMS sample.

Sample	${\bar{M}}_{n}^{PS\;}$ (kg mol^−1^) ^a^	${\bar{M}}_{n}^{PDMS\;}$ (kg mol^−1^) ^a^	${\bar{M}}_{n}^{total\;}$ (kg mol^−1^) ^a^	*Đ* ^b^	*f*_PDMS_ *^v^* ^c^
PS precursor	51			1.03	
PS-*b*-PDMS	51	35	86	1.05	0.42

^a^ Number average molecular weight of PS and PDMS determined by membrane osmometry (MO). ^b^ Dispersity (*Đ*) measured by size exclusion chromatography (GPC). ^c^ Volume fraction of PDMS (*f*_PDMS_*^v^*) in the PS-*b*-PDMS as calculated from ^1^H NMR based on *ρ*_PS_ = 1.04 g/cm^3^, *ρ*_PDMS_ = 0.965 g/cm^3^.

## Data Availability

Not applicable.

## Supplementary Materials

The following supporting information can be downloaded at: https://www.mdpi.com/article/10.3390/polym14091891/s1, Figure S1: DSC analysis of PBA-*b*-PMMA. (a) The glass transition temperature of PBA. (b) Glass transition temperature of PMMA. (c) DMA analysis of PBA-*b*-PMMA with a glass transition temperature of PBA. (d) DSC analysis of epoxy resin with 0%, 5%,10%,15%, and 20% of PBA-*b*-PMMA.; Figure S2: (a) TEM micrograph of solution-cast PS-b-PDMS using toluene as solvent; (b) Corresponding 1D SAXS profile; (c) TEM micrograph of solution-cast PS-b-PDMS using chloroform as solvent; (d) Corresponding 1D SAXS profile; Figure S3: Three-dimensional TEM reconstruction images of (a) gyroid- and (b) diamond-structured epoxy resins in PS matrix from templated polymerization; Figure S4: Load–displacement curve of intrinsic epoxy resin (black) and intrinsic epoxy resin with 5% of PBA-*b*-PMMA (red); Figure S5: Load–displacement curve of Gyroid and diamond- structured epoxy resin without modifier; Table S1: Characterization of PBA-*b*-PMMA.
