# Supplementary material for: Block Copolymer Modified Nanonetwork Epoxy Resin for Superior Energy Dissipation
Source: Polymers (Basel). 2022 May 5;14(9):1891. doi: 10.3390/polym14091891 (PMC9105528; doi:10.3390/polym14091891)
Supplement: Supplementary file 1 [file polymers-14-01891-s001.zip › polymers-1681818-supplementary.pdf]

Block Copolymer Modified Nanonetwork Epoxy Resins for Superior Energy Dissipation

Suhail K. Siddique[†], *Hassan Sadek*[†], *Tsung-Lun Lee*[†], *Cheng-Yuan Tsai*[§], *Shou-Yi Chang*[§], *Hsin-Hsien Tsai*[‖], *Te-Shun Lin*[‖], *Gkreti-Maria Manesi*[⊥], *Apostolos Avgeropoulos*[⊥] *Rong-Ming Ho*[*†]

[†]Department of Chemical Engineering, National Tsing Hua University, Hsinchu 30013, Taiwan.

[§]Department of Material Science and Engineering, National Tsing Hua University, Hsinchu 30013, Taiwan.

[‖]Kaohsiung Factory R&D Department, Chang Chun Plastics Co Ltd, Kaohsiung 81469, Taiwan.

[⊥]Department of Materials Science Engineering, University Campus, University of Ioannina, Ioannina 45110, Greece.

[¥]Faculty of Chemistry, Lomonosov Moscow State University (MSU), GSP-1, 1-3 Leninskiye Gory, 119991 Moscow, Russia

Charecterization of BCP modifier

The acrylic acrylic-based block copolymer under the trade name of D51N®, namely poly(butyl acrylate)-*b*-poly(methylmethacrylate), was used as a modifier for epoxy resins. The structure of these block copolymers can be tuned to give excellent toughening to a wide range of epoxy resins systems without sacrificing strength or thermal properties. A systematic study of the chemical and thermal properties of the modifier has been conducted. The dispersity (Đ) index of the PBA-*b*-PMMA BCP was determined, giving a value of 1.61. The fundamental study with respect to the toughening effect of PBA-*b*-PMMA is strongly related to the basic understanding of the block copolymer physical properties analysis up to thermal characterization methods such as differential scanning calorimetry (DSC). As shown in **Figure S1a**, the glass transition temperature of the soft segment (PBA) is approximate -35°C, whereas the hard segment PMMA **(Figure S1b)** has a T_g value at approximately 87 °C. Note that, the T_g of the soft segment is important for the toughening mechanism of DGEBA. Moreover, the hard segment should have affection with epoxy resins to adopt the proper self-assembled morphology. The T_g of the soft segment (PBA) was confirmed by the dynamic mechanical analysis (**Figure S1c**). Note that, here the tan δ shows that the actual T_g of PBA is approximate -37°C, which can be considered sufficient for toughening. With the use of the PBA-*b*-PMMA, the PMMA block leads to the compatibility of the epoxy resin whilst the PBA block provides an immiscible soft rubber phase for toughening. The PBA-*b*-PMMA can be easily dissolved in typical diglycidyl ether of bisphenol A (DGEBA) resins with the application of heat and a low amount of shear. When dissolved in DGEBA resin, the PBA-*b*-PMMA can self-assemble into nanostructures. The PMMA block is associates with the epoxy resin, forming an effective shell surrounding an immiscible PBA core. The analysis of glass transition temperature change for epoxy resins after

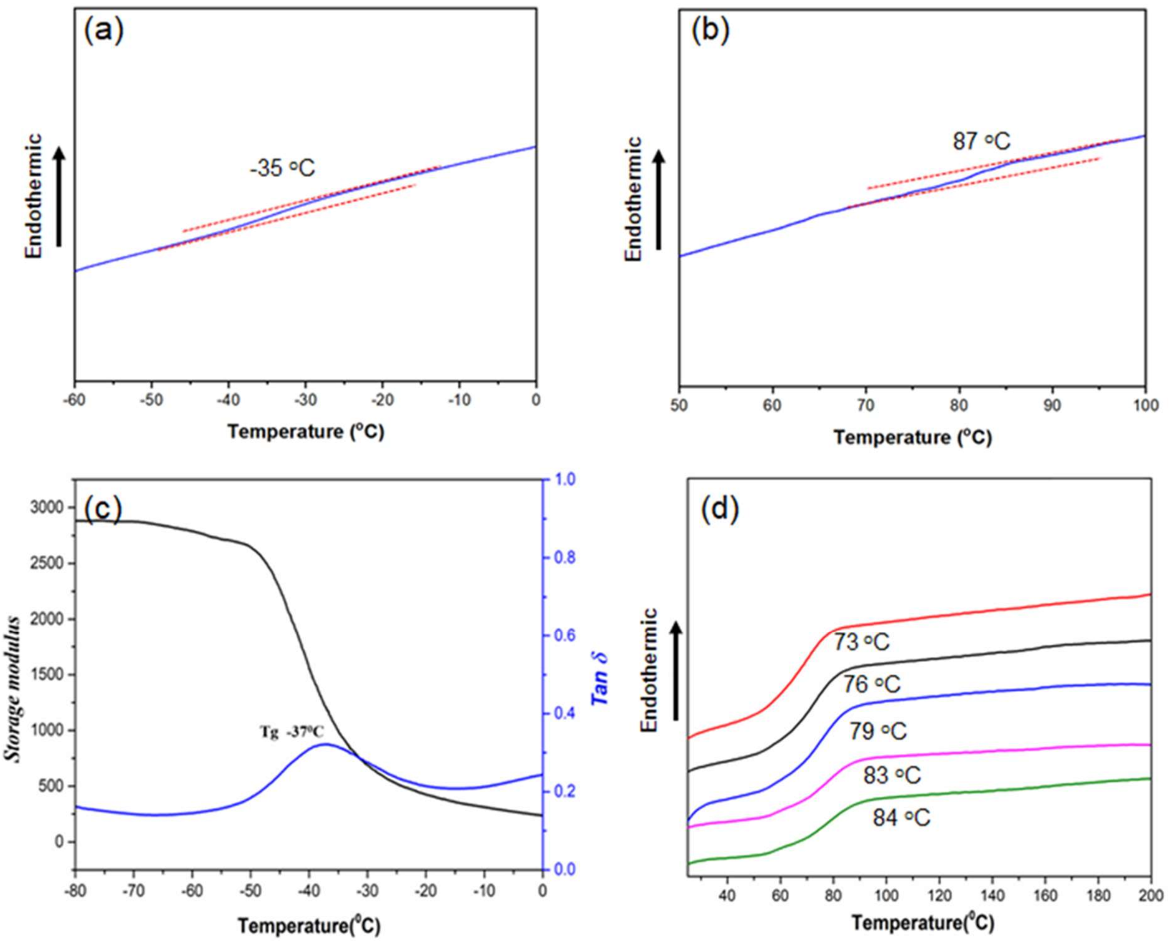

Figure S1. DSC analysis of PBA-*b*-PMMA. (a) The glass transition temperature of PBA. (b) The glass transition temperature of PMMA. (c) DMA analysis of PBA-*b*-PMMA with a glass transition temperature of PBA. (d) DSC analysis of epoxy resins with 0%, 5%,10%,15%, and 20% of PBA-*b*-PMMA.

preparing the BCP toughened epoxy resins was investigated using DSC. By taking advantage of templated polymerization, well-ordered gyroid- and diamond-structured epoxy modified with D51N® can be prepared. Note that, the thermal properties of epoxy resins are not affected substantially from the addition of the modifier. We analyze the T_g of pure epoxy resins, with 5%,10%,15%, and 20% of modifier; here the T_g of pure epoxy resins is approximately 84°C and the T_g is reduced to 73°C while loading the BCP with 20%of modifier (**Figure S1d**). **Table S1**

shows the characterization of PBA-*b*-PMMA; the table was added in the revised manuscript (see supporting information).

Table 1. Characterization of PBA-*b*-PMMA.

Sample	f_{PBA}^{v}	$\bar{M}_n^{total}$ (g mol^{-1})	$Đ$	$\bar{M}_w^{total}$ (g mol^{-1})
PBA-*b*-PMMA	0.32	32,800	1.34	44,300

Number average molecular weight and weight average molecular weight of PBA-*b*-PMMA determined by Gel permeation chromatography (GPC).
Dispersity ($Đ$) was measured by Gel permeation chromatography (GPC). The volume fraction of PBA (f_{PBA}^{v}) in the PBA-*b*-PMMA calculated from ^{1}H NMR based on ρ_{PBA}= 1.3 g/cm^3, ρ_{PMMA} =1.18 g/cm^3.

The characterization of molecular weight of PBA-*b*-PMMA block copolymer was carried out using gel permeation chromatography (GPC) and proton nuclear magnetic resonant (^{1}H NMR). As shown above, the number average molecular weight and weight average molecular weight of the PBA-*b*-PMMA were determined as 32,800 g/mol and 44,300 g/mol, For the molecular weight determination using GPC, a calibration curve by using PS standards in a common solvent (THF) was carried out. Note that the size of the spherical micelle can be controlled by molecular weight as mentioned by Bates *et. al.*[1] As a result, the average spherical micelle size will be dependent upon molecular weight; here, the PBA-*b*-PMMA used in this study is a commercial sample from Arkema® D51N with a micelle size of approximately 15nm which is generally regarded as one of the best toughen modifiers for epoxy resins. To control the size of the spherical micelle, one easy way is to tune the molecular weight of PBA-*b*-PMMA**.**

Preparation of well-ordered nanoporous template.

The lamellae-forming PS-*b*-PDMS diblock copolymer was dissolved in selective solvents including toluene and chloroform at a concentration of 10 wt% in a vial and then sealed well

by aluminum foil having punch holes for the evaporation of the solvent. The as-cast sample was acquired by drying in ambient conditions, and then further dried in a vacuum oven at 60°C to remove residual solvent. The formation mechanisms of network phases from the self-assembly of lamellae-forming PS-*b*-PDMS at which double gyroid could be obtained by using toluene for solution casting (**Figure S2**); the formation of double diamond could be achieved by using chloroform. Moreover, the affinity of PS to PS-selective solvent results in the reduction on the

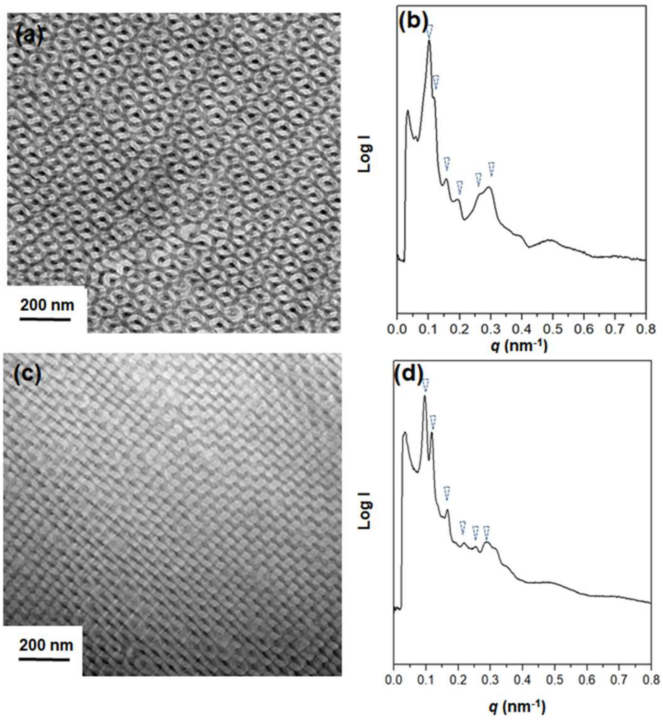

Figure S2. (a) TEM micrograph of solution-cast PS-*b*-PDMS using toluene as solvent; (b) Corresponding 1D SAXS profile;(c) TEM micrograph of solution-cast PS-*b*-PDMS using chloroform as solvent; (d) Corresponding 1D SAXS profile.

volume fraction of PDMS which brings curvatures to the flat interfaces (lamellar morphology) that generates network or cylindrical structures. Subsequently, with the high etching selectivity between the PS and PDMS segments, the nanoporous PS template can be obtained from the self-assembled PS-*b*-PDMS using HF aqueous solution (HF/H_2O/methanol =0.5/1/1 by volume) to etch the PDMS block. After complete degeneration of the PDMS block followed by rinsing with

a mixture of distilled water and methanol, well-ordered nanoporous PS templates with gyroid- and diamond-structured nanochannels can be obtained, giving PS templates having gyroid- and diamond-structured porous textures with approximately equivalent porosity for templated polymerization.

The geometry of the forming modified nanonetwork polymers is the central issue for the character of mechanical properties. The unit cell (*a*) and the interdomain distance (*d*) as determined from SAXS, the strut (ligament) thickness (*t*) as determined from the reconstruction image of 3D TEM **(Figure S3)** for the forming nanonetwork structures. Here, the PS-*b*-PDMS contains 38 vol% of PDMS for double gyroid; after replacement of the PDMS with epoxy, the nanonetwork epoxy in the PS matrix gives a = 155.6 nm and d = 63.5 from the SAXS results and t = 26.0$\pm$3.0 nm from the reconstruction image by electron tomography. By contrast, the one with double diamond gives a = 92.54nm and d = 65.4 from the SAXS results and t = 23.0$\pm$3.0 nm. Note that the filling ratio (filling fraction) of the polymeric resins synthesized from templated polymerization is a function of the strut (ligament) thickness. Moreover, the average size of the spherical micelle will be approximately 15nm based on the morphological analysis from TEM. Unfortunately, it is not feasible to recognize the dispersed micelle in the epoxy due to the lack of staining agent for the required contrast in the nanonetwork of epoxy; yet, it should be reasonable to have the smaller micelle forming in the epoxy strut with much larger diameter.

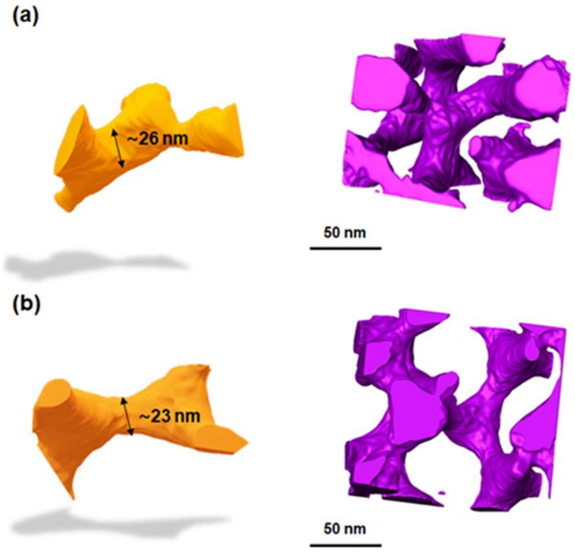

Figure S3. Three-dimensional TEM reconstruction images of (a) gyroid- and (b) diamond-structured epoxy resins in PS matrix from templated polymerization.

Energy Dissipation Calculation

All the samples used in this study were in the thin-film state with thickness of approximately 5μm micrometer for nanoindentation test; in fact, this work aims to demonstrate the effect of deliberating structuring on the mechanical performance of thin-film samples in the application of nanoMEMS process. Regarding the tensile tests, it is usually carried out on large sample size (3x3cm^2) for commodity applications to understand fracture toughness. The nanoindentation measurements were carried out using a nanoindenter (TI900 TriboIndenter, Hysitron Inc.) for the examination of at least four locations to test the gyroid- and diamond-structured thermosets. The load (up to a maximum of 500 μN) was applied by a diamond spherical indenter with 2 μm diameter at the same rate of loading and unloading (60 μN/sec) (**Figure S4 and S5**).

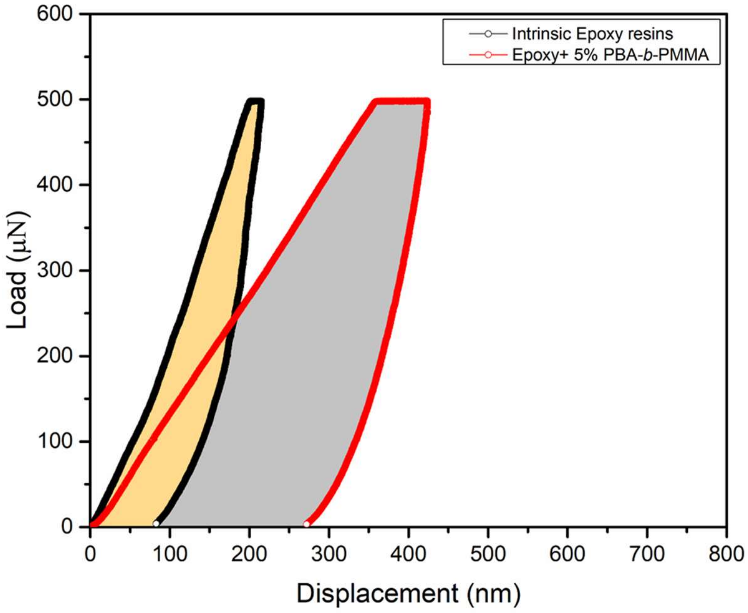

Figure S4. Load-displacement curve of intrinsic epoxy resins (black) and intrinsic epoxy resins with 5% of PBA-*b*-PMMA (red).

Here, the effect of structure on energy dissipation capabilities has been already reported in our previous publication.[2] With the well-ordered network, it is capable to dissipate the applied stress through struts in the gyroid- and diamond-structured resins. The fabricated nanonetwork resins with diamond structure (tetrapod) possess higher energy dissipation than gyroid-structured (tripod). Accordingly, specific nanonetwork structures with ordering periodically improve the material toughness by dissipating energy through the well-connected nanonetworks. To further enhance the energy dissipation of the nanonetwork epoxy as a follow-up work, a facile approach is to introduce a block copolymer-based (BCP) modifier, poly(butyl acrylate)-*b*-poly(methyl methacrylate) (PBA-*b*-PMMA), to the well-ordered nanonetwork epoxy resins. For the homogeneous, non-structured epoxy resins, the calculated energy dissipation is $0.02\underline{+}0.002$ nJ; note that, the epoxy resins with the highest crosslinking density is nearly 90% elasticity at this loading, as evidenced by the unloading curve with significant retracting. By contrast, after being modified with 5% of PBA-*b*-PMMA, a five times higher energy dissipation, equal to

0.09±0.004 nJ,than the intrinsic brittle epoxy resins is obtained with the plastic deformation mode. After removal of the PS template, the energy dissipation of gyroid epoxy without modifier further increases to 0.12±0.008 nJ. which is approximately six-times the intrinsic epoxy resin. Most interestingly, the diamond-structured epoxy resins show a significant escalation in the indentation depth which thus gives outstanding energy dissipation of approximately 0.2±0.008 nJ.

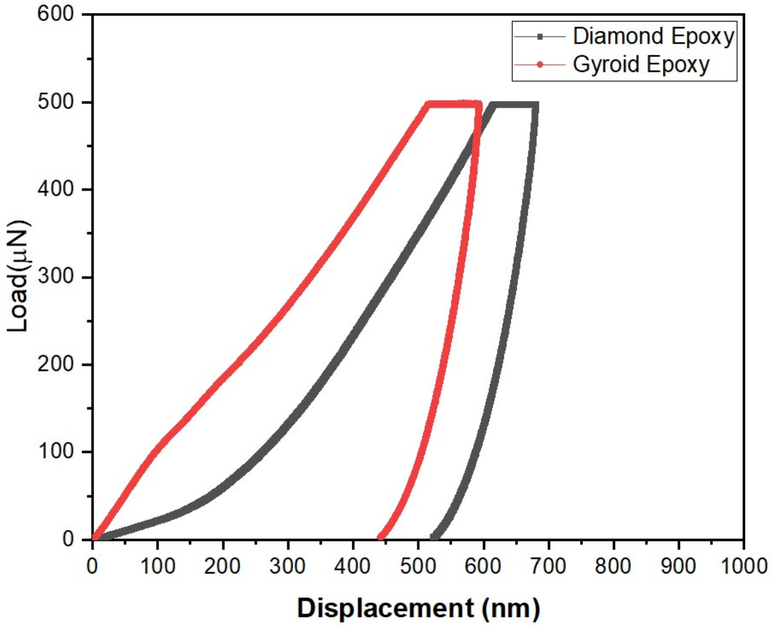

Figure S5. The load-displacement curve of gyroid and diamond-structured epoxy resins without modifier.

References

1. Dean, J. M.; Lipic, P. M.; Grubbs, R. B.; Cook, R. F.; Bates, F. S., Micellar structure and mechanical properties of block copolymer-modified epoxies. *Journal of Polymer Science Part B: Polymer Physics* **2001,** *39* (23), 2996-3010.
2. Siddique, S. K.; Lin, T.-C.; Chang, C.-Y.; Chang, Y.-H.; Lee, C.-C.; Chang, S.-Y.; Tsai, P.-C.; Jeng, Y.-R.; Thomas, E. L.; Ho, R.-M., Nanonetwork Thermosets from Templated Polymerization for Enhanced Energy Dissipation. *Nano Letters* **2021,** *21* (8), 3355-3363.
